# Effect of Cerium Salt and Zeolite Particle-Doped Silane Film on Corrosion Resistance of Epoxy Coating on 7N01 Aluminum Alloy

**DOI:** 10.3390/ma18174026

**Published:** 2025-08-28

**Authors:** Lin Sun, Sha Peng, Han Wang, Xinyu Lv, Jianguo Tang, Ming-An Chen

**Affiliations:** 1 School of Materials Science and Engineering, Central South University, Changsha 410083, China; sunlin0218@163.com (L.S.); m18245174207@163.com (S.P.); csuwanghan@163.com (H.W.); jgtang@csu.edu.cn (J.T.); 2 Northeast Light Alloy Co., Ltd., Harbin 150060, China; crrcfc123@163.com; 3 CRRC Qingdao Sifang Co., Ltd., Qingdao 266000, China

**Keywords:** aluminum alloy, epoxy coating, zeolite, cerium nitrate, silane

## Abstract

In order to enhance the anti-corrosion property of epoxy coatings on 7N01 aluminum alloy, cerium nitrate and zeolite particles were incorporated into a bis-(triethoxysilylpropyl)tetrasulfide (BTESPT) silane solution to pretreat the substrate. Scanning electron microscopy (SEM) and an electronic probe microanalyzer (EPMA) were used to characterize the morphology and chemical composition of the composite silane film. The corrosion performances of the epoxy coatings were evaluated by potentiodynamic polarization and electrochemical impedance spectroscopy (EIS) and based on the morphology and chemical composition of the interfacial region after salt spray tests. The thickness of the composite silane film at 5% BTESPT doped with 5 × 10^−3^ M cerium nitrate and 0.5 g/L zeolite particles was about 2.1 μm. The composite silane film can provide active protection to the substrate surface beneath the epoxy coating. It promotes the impedance value of the coating at 10^−2^ Hz by two to three orders of magnitude and greatly lessens the interfacial region corrosion between the coating and the substrate. This effect can be ascribed to the strong barrier effect of the composite silane film and cerium ions released from the silane network and the zeolite particles.

## 1. Introduction

7N01 aluminum alloy is widely applied for components of high-speed trains due to its light weight, high strength, high fracture toughness and resistance to fatigue, satisfactory welding properties, and excellent extrusion properties [1,2,3]. However, due to its complex chemical composition, numerous intermetallic particles, such as MgZn_2_ (η phase), AlFeMnSi, Al_3_Zr, etc., are formed in the Al matrix. These particles have different electrochemical activities, showing high potential difference between them and the Al matrix, which leads to galvanic corrosion when subjected to aggressive environments [1,2,3].

In order to enhance anti-corrosion properties, various kinds of protective coatings are adopted to separate substrates from corrosion-prone environments. Among them, epoxy-based coatings are widely used due to their outstanding protection of the metal substrates against corrosion through excellent barrier effects against the infiltration of corrosive electrolytes onto the metal surface and mechanical property [4,5,6,7].

However, for epoxy coatings, when a mixture of epoxy and a curing agent like polyamide is applied to a metal substrate, the curing agent is preferentially adsorbed by metal oxide on the surface of the metal substrate [8,9,10,11]. In exposed environments, water and accompanying aggressive media can penetrate rapidly along the interface and accumulate in it, resulting in decline of the adhesive strength of the epoxy coating to the metal substrate and corrosion of the metal surface in the region of reduced adhesion [4,5,8,9,10,11].

Thus, in order to improve the adhesive strength and corrosion protection of the metal surface underneath organic coatings, efforts have been made through modifications to epoxy coatings by the addition of nano/micro particles into the resin or forming layered coatings and surface pretreatment of the metal substrates prior to the coating process. Recently, Muresan et al. reported that epoxy resin/zinc hybrid coatings applied on steel by electrodeposition have good corrosion resistance [12]. Bogatu et al. reported the effectiveness of protective layered coatings formed by epoxy primer and then polyurethane paint with kreutzonit particles to prevent steel corrosion under marine conditions [13]. As for the approach to surface pretreatment of metal substrates, in recent decades, several effective methods, including chromate conversion coatings (CCCs), phosphating, anodization, etc., have been adopted to change the surface states of aluminum substrates to strengthen the interface between aluminum and epoxy [4,5,8,9,10,11]. Among these approaches, chromate conversion coatings (CCCs)/chromating is an effective and widely used metal finishing approach for many years. However, it is banned or strongly restricted due to the hazardous hexavalent chromium compounds associated with it.

To this end, van Ooij et al. pioneered organosilanes as a promising replacement for hexavalent chromating and phosphating pretreatment processes in metal finishing [14,15,16,17,18,19,20,21]. The silanes of aminopropyltriethoxysilane (APS), tetraethoxysilane (TEOS), methyltriethoxysilane (MTES) and γ-glycidoxypropyltrimethoxysilane (γ-GPS), and several kinds of bis-silanes, such as bis-(trimethoxysilylpropyl)amine (Bis-amino), bis-1,2-(triethoxysilyl)ethane (BTSE) and bis (triethoxysilylpropyl)tetrasulfide (BTESPT), and mixtures of two kinds of silanes as BTESPT/bis-amin and bis-amino/vinyltriacetoxy silane (VTAS), have been attempted to enhance the anti-corrosion property of steels, aluminum alloys, Zn-coated steels and Cu alloys, etc. [14,15,16,17,18,19,20,22,23,24,25]. Silane film can strengthen the adhesive bonding between protective organic coatings and metal substrates and enhance the anti-corrosion ability of organic coatings on metal substrates [14,15,16,17,18,19,20,21,22,23,24,25]. Fedel et al. [26] improved the anti-corrosion properties of epoxy-polyester powder coating on galvanized steel by a barrier effect against water and oxygen through sol–gel pre-treatment of the substrate with the silane compounds GPS, TEOS, and MTES. Romanoa et al. [27] reported that the barrier effects and filiform corrosion resistance of the cataphoretic epoxy coating on 6016 aluminum alloy was improved by sol–gel pretreatment of the substrate with the silane compounds of GPS, TEOS, and MTES. Our previous work [28] showed that the pretreatment of 2A12 aluminum alloy sheet with BTESPT silane is effective to lessen corrosion of the substrate underneath the epoxy coating. These published results show that silane-based pretreatment is a promising technology to replace chromates and phosphates in metal finishing.

However, silane films do not have active electrochemical behavior, and aggressive media can reach the interfacial region through defects such as micro pores, cracks and low-cross-linked zones in the thin silane film [21]. Many researchers have attempted to provide silane films with active self-healing for improvement of long-term corrosion protection [29,30,31,32,33,34]. Rare earth salts that contain Y^3+^, La^3+^, Pr^3+^, and Nd^3+^ ions, especially Ce^3+^, are corrosion inhibitors. Therefore, incorporation of these rare earth salts into silane films to improve corrosion resistance has been attempted [10,11,14]. Baharmi et al. [35] reported that the anti-corrosion property of a Si/Zr sol-gel hybrid coating on 6061 aluminum alloy was improved by doping cerium nitrate in it. Cabral et al. [36] reported that the pretreated silane film by BTESPT solution doped with cerium nitrate had longer lifetime protection to corrosion of dip galvanised steel and 2024-T3 Al substrates. Shi et al. [37] reported that the addition of 1 × 10^−3^ M Ce(NO_3_)_3_ into silane solution of GPTMS and TMOS significantly improved the corrosion resistance of the sol-gel coating on 2024-T3 aluminum alloy. Naderi et al. [30] reported that the stability of pure Al interface was improved by adding of cerium cations into silane sol-gel coating. Unfortunately, the corrosion inhibitors are prone to be uncontrolled fast leaking [38,39,40,41].

Zeolite particles, microporous cage-structured aluminosilicate crystals, have large amounts of nanoporous channels and high chemical affinity for adsorption of a variety of cations. These properties confer the zeolite particles can be used as a reservoir to adsorb the rare earth ions like Ce^3+^ and have been adopted to prepare different composite silane films on metals surface by adding Ce-enriched zeolite into the silane solution [38,39,42]. The analysis by Energy-dispersive X-ray Fluorescence (EDXRF) demonstrates that cerium concentration in the Ce-enriched zeolite particles prepared by ion exchange is 14.6 wt.% [39]. When local corrosion occurs Ce^3+^ can be released from zeolite particles to the corrosion region to slow down the corrosion process [38,39]. In addition, zeolite particles are nontoxic, thermal and chemical stable in various aggressive conditions. Dias et al. [38,39] reported that the anti-corrosion performance of a sol-gel hybrid coating was enhanced by doping of cerium-enriched zeolite (NaX) into the hybrid solution of a glycidoxypropyl-trimethoxy-silane (GPTMS)-based alkosol and an alkosol containing zirconium tetrapropoxide. Zheludkevich et al. [40] demonstrated that the sol-gel film of TEOS and GPTMS doped with zirconia nanoparticles and cerium nitrate, or cerium nitrate doped oxide nanoparticles on 2024-T3 Al substrate led to promotion of the anti-corrosion property. The zirconia particles act as nanoreservoirs to prolong release of cerium ions. Urša Tiringer et al. [41] reported that incorporation of colloidal SiO_2_ and cerium nitrate into a GPTMS/TEOS based sol-gel coating on 7075-T6 Al substrate increased its anti-corrosion property.

In our previous work [43], it was shown that for corrosion protection to 7N01 aluminum sheets the proper bis-[triethoxysilylpropyl]tetrasulfide silane (BTESPT) silane volume was 5% and the optimum cerium nitrate added into the silane solution was 5 × 10^−3^ M. Cabral et al. [36] indicated that the silane film, doped with 1 × 10^−3^ M cerium nitrate in BTESPT solution, on dip galvanized steel and AA2024-T3 aluminum substrates promoted the longer lifetime protection to corrosion.

Therefore, in this work, different contents of zeolite particles were added into 5% silane solution incorporated by 5 × 10^−3^ M cerium nitrate to form a composite film on 7N01 Al substrates prior to epoxy coating process. The work aims to evaluate the anti-corrosion property of epoxy coatings on 7N01 substrates without and with this pretreatment. The corrosion properties were examined by potentiodynamic polarization, electrochemical impedance spectroscopy (EIS), immersion and salt spray tests. Scanning electron microscopy (SEM), electronic probe microanalyzer (EPMA) were used to characterize morphology and chemical composition of the composite silane films and the interfacial region between the epoxy coating and the substrate.

## 2. Experimental Methods

### 2.1. Materials and the Coating Process

#### 2.1.1. 7N01 Substrates

The used 7N01 aluminum alloy substrates were rolled sheets with a thickness of 3 mm (wt.: Zn 4.6%, Mg 1.7%, Mn 0.55%, Cu 0.16%, Cr 0.2%, Si 0.2%, Zr 0.15%, balance Al). All the sheet substrates were boiled in boiling water for 20 min, mechanical polished by SiC paper up to grit 1500, rinsed by tap water. Then, the substrates were immersed in 0.3M NaOH solution for two minutes, cleaned with deionized water and drying at room temperature. Finally, the pretreated substrates were ultrasonic cleaned in ethanol and deionized water for a few minutes, dry in air before used.

#### 2.1.2. Preparation of Silane Solution with Cerium Nitrate and Nano Zeolite Particles

The bis-[triethoxysilylpropyl]tetrasulfide silane ((H_5_C_2_O)_3_Si(CH_2_)_3_S_4_(CH_2_)_3_Si(OC_2_H_5_)_3_) solutions were prepared by dissolving 5% (*v*/*v*) of the silane in ethanol 90% (*v*/*v*) and deionized water 5% (*v*/*v*), then 5 × 10^−3^ M cerium nitrate and 0.25 g/L, 0.5 g/L and 2.5 g/L zeolite particles (ZSM-5 with the average diameter < 500 nm, prepared by Zhuoran environmental protection Co., Ltd., Dalian, China) were added into the silane solutions, respectively. The mixed silane solutions were stirred for 30 min before use.

#### 2.1.3. Preparation of the Composite Silane Film and Top-Coated Epoxy Coating

The above pretreated Al substrates were immersed in the prepared silane solutions for 3 min. Then, these substrates were cured in an oven at 120 °C for 2 h to form a composite silane film on their surfaces.

The used epoxy coating is a two components epoxy-polyamide material commercialized by Zhongshan Daoqum Chem. Com., Zhongshan, China (epoxy EP160 and polyamide based curing agent A-017 with a weight ratio of 6:1 according to the manufacturer recommendations). The epoxy was mixed with solvent (25 vol.% n-butanol and 75 vol.% xylene) by stirring to form the mixture with an amount of 39 mass% of solvent-mixture. The resin mixture was spray coated onto the substrates. The spray coating process should be controlled to produce the comparable surface quality and thickness on all samples. Then, the spray-coated samples were cured for one week at room temperature. As can be seen in the following by SEM the thickness of the spray prepared epoxy coating on all samples are about 70 μm.

### 2.2. Characterization

The features of morphology and chemical composition of different samples were examined by SEM and EDS analysis (ZEISS EVO MA 10 (ZEISS, Oberkochen, Germany) and MIRA3 LMH (TESCAN, Brno, Czech Republic) operated at 20 kV). The distribution of typical elements on surface and cross-sectional of the samples was performed using the Electron Probe Microanalyzer (JXA-8230, JEOL, Tokyo, Japan).

Soaking tests were performed in 3.5 wt.% NaCl solution at room temperature. Salt spray tests were carried out by exposing to 5 wt.% NaCl solution at 35 ± 2 °C according to ASTM B 117 standard [44].

The potentiodynamic polarization and electrochemical impedance spectroscopy (EIS) tests were carried out in 3.5 wt.% NaCl aqueous solution at ambient temperature by CHI Model 660E electrochemical work station. The tested sample was the working electrode, and its exposed area to the solution is 1 cm^2^. The polarization tests were scanned from −1.4 V to −0.2 V at 0.002 V/s. The EIS tests were performed at the open circuit potential. The frequency range was from 10^−2^ Hz to 10^5^ Hz. The applied AC sine wave amplitude was 10 mV.

## 3. Results and Discussion

### 3.1. Effect of Zeolite Particles on the Surface Pretreated Substrates

**Figure 1:**
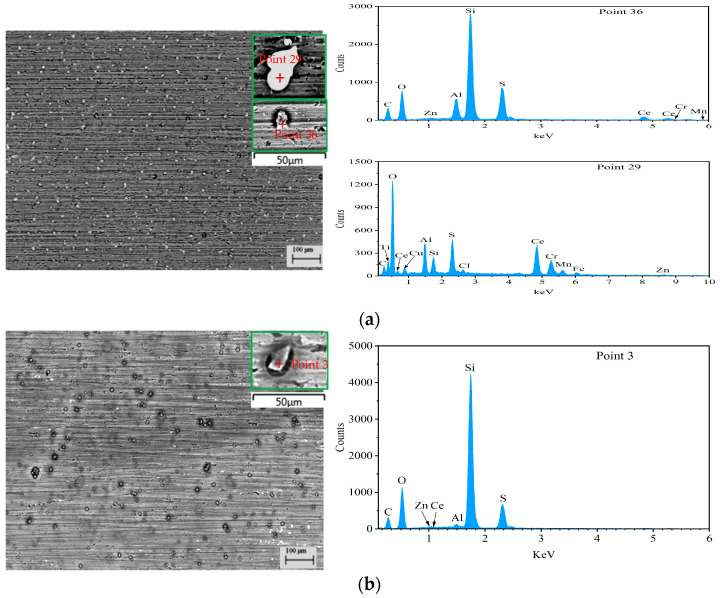

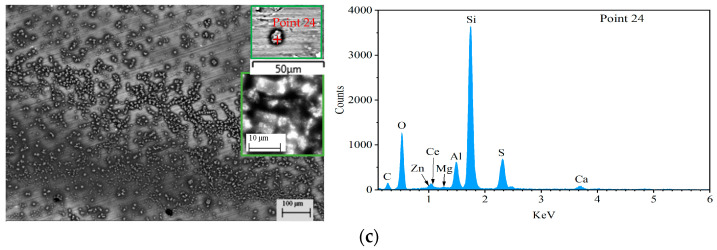
Surface SEM morphologies and EDS spectra of marked points of the composite silane films by 5% BTESPT doped with 5 × 10^−3^ M cerium nitrate and different contents of zeolite particles, (**a**) 0.25 g/L, (**b**) 0.5 g/L and (**c**) 2.5 g/L.

Figure 1 shows surface SEM morphologies of the prepared composite silane films on 7N01 substrates by 5% BTESPT doped with 5 × 10^−3^ M cerium nitrate and different contents of zeolite particles. For the case with 0.25 g/L zeolite particles, a lot of white particles can be observed, as shown in Figure 1a. The EDS spectrum of point 29 and the chemical composition in Table 1 show that it contains 28.79 wt.% Ce, indicating it is cerium oxides or hydroxides [29,30,33,36,37,38]. In addition, gray particles, marked by 36, can also be found. According to its EDS spectrum and the chemical composition in Table 1, it contains high contents of Si and O, confirming it is a zeolite particle doped with Ce. When the zeolite particles increased to 0.5 g/L, a lot of gray particles can be seen in Figure 1b (compared with Figure 1a). These are zeolite particles doped with Ce (see point 3 in Table 1). When the zeolite particles increased to 2.5 g/L, a great number of gray particles can be seen in Figure 1c. Also, they are the zeolite particles doped with Ce (see point 24 in Table 1). However, many agglomerated zeolite particles are aggregated together, as shown by magnification in Figure 1c, which will create defects such as micro cracks and micro voids between the agglomerated zeolite particles [45,46,47].

**Figure 2:**
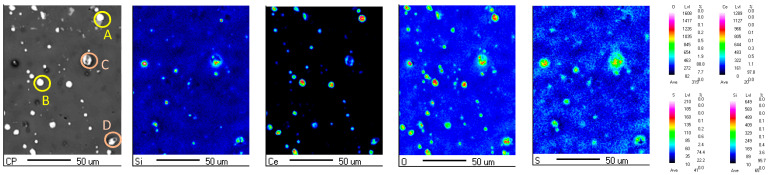
Surface EPMA elemental mapping of the composite silane film by 5% BTESPT doped with 5 × 10^−3^ M cerium nitrate and 0.5 g/L zeolite particles.

Figure 2 presents the EPMA surface elemental mapping of the pretreated composite silane film on 7N01 substrate by 5% BTESPT doped with 5 × 10^−3^ M cerium nitrate and 0.5 g/L zeolite particles. As for the particles A and B, they contain high Ce and O, indicating that they are cerium oxides or hydroxides independently precipitated in the composite silane film [36,37,38]. While for the particles C and D, they contain high O, Si and Ce, indicating that they are zeolite particles with Ce adsorbed.

**Figure 3:**
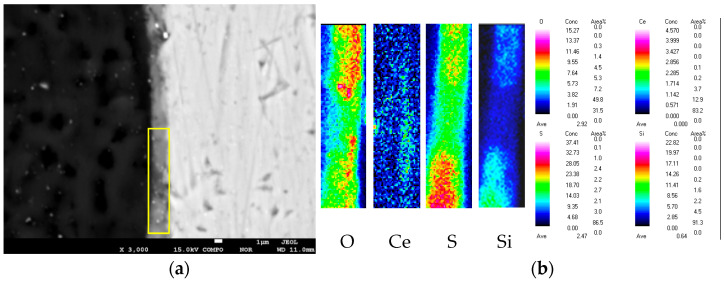
(**a**) Cross-sectional morphology of the composite silane film by 5% BTESPT doped with 5 × 10^−3^ M cerium nitrate and 0.5 g/L zeolite particles and (**b**) EPMA elemental mapping.

Figure 3 gives the cross-sectional morphology of the composite silane film by 5% BTESPT doped with 5 × 10^−3^ M cerium nitrate and 0.5 g/L zeolite particles and the EPMA elemental mapping. The thickness of the composite silane film, determined by Figure 3a, is about 2.1 μm, while for the pure silane films on metal substrates, their thicknesses are below 1 μm, generally around 0.2–0.5 μm [36].

**Figure 4:**
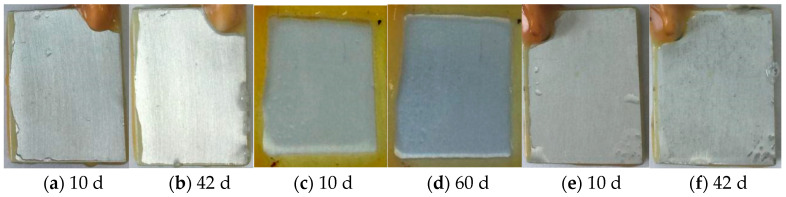
Surface images of the silane films by 5% BTESPT doped with 5 × 10^−3^ M cerium nitrate and different contents of zeolite particles during immersion in 3.5 wt% NaCl solution, (**a**,**b**) 0.25 g/L, (**c**,**d**) 0.5 g/L and (**e**,**f**) 2.5 g/L.

Figure 4 shows the surface images of the pretreated composite silane films on 7N01 substrates by 5% BTESPT doped with 5 × 10^−3^ M cerium nitrate and different contents of zeolite particles during immersion in 3.5 wt.% NaCl solution for different time. Our previous work [43] has shown that local corrosion occurred after 28 days of immersion for the sample pretreated by the silane film doped with 5 × 10^−3^ M cerium nitrate. For the sample pretreated by the silane film doped with 5 × 10^−3^ M cerium nitrate and 0.25 g/L zeolite particles, local corrosion can be observed after 42 days of immersion, as shown in Figure 4b, suggesting that addition of zeolite particles improves the corrosion protection of the silane film. When the zeolite particles increase to 0.5 g/L, almost no any change can be observed on surface of the sample even after 60 days immersion, as shown in Figure 4d, demonstrating that excellent corrosion protective property of the composite silane film. However, when the zeolite particles increase to 2.5 g/L, obviously local corrosion occurred only after 10 days immersion as shown in Figure 4e, indicating that incorporation of higher zeolite particles into the silane film leads to negative impact on protective property and therefore causes worse corrosion protection. This can be ascribed to aggregation of the agglomerated zeolite particles as shown in Figure 1c, which causes formation of micro defects between the agglomerated zeolite particles [45,46,47]. These defects will favor penetration of the aggressive media through the film and decrease its barrier properties [46,47]. Therefore, the following work will focus on corrosion performances of the epoxy coatings on 7N01 substrates without and with pretreatment by 5% BTESPT doped with 5 × 10^−3^ M cerium nitrate and 0.5 g/L zeolite particles.

### 3.2. Corrosion Performance of the Epoxy Coated Substrates

**Figure 5:**
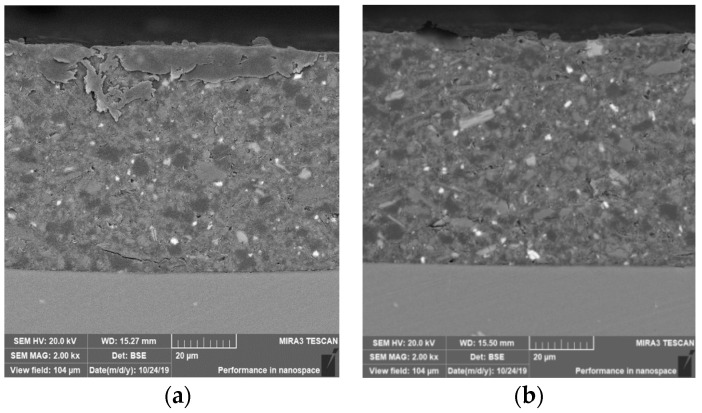
Cross-sectional morphologies for the samples with epoxy coating on (**a**) 7N01 substrate and (**b**) the 7N01 substrate pretreated by the silane film doped with 5 × 10^−3^ M cerium nitrate and 0.5 g/L zeolite particles.

Figure 5 shows the cross-sectional morphologies for the sample with epoxy coating on blank 7N01 substrate and the sample with epoxy coating on 7N01 substrate pretreated by the composite silane film doped with 5 × 10^−3^ M cerium nitrate and 0.5 g/L zeolite particles. It can be seen that both of the interfacial regions are flat without apparent irregularities. No cracks can be observed at the interfacial regions, demonstrating that the adhesion between the epoxy coatings and the substrates are in well adhered state in both cases to endure the mechanical cutting and polishing processes. The thickness of the epoxy coatings on the substrates by SEM measurement is about 70 μm.

#### 3.2.1. Polarization Tests

**Figure 6:**
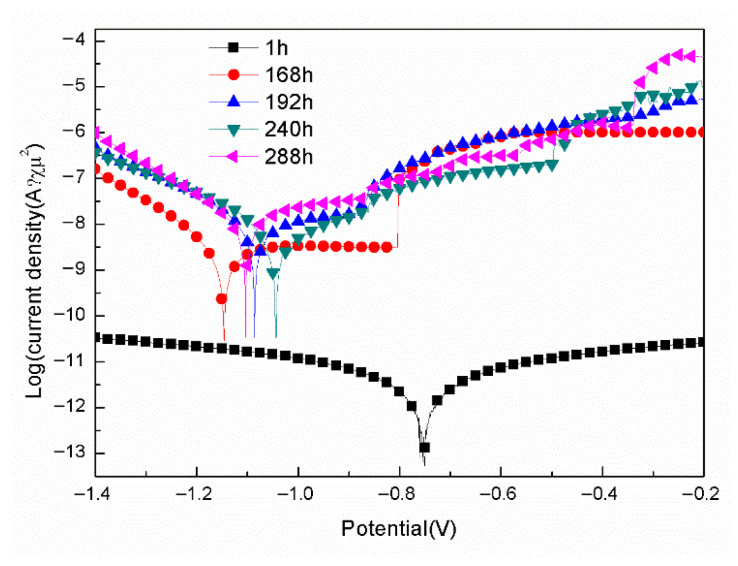
Polarization curves for the samples with epoxy coatings on the blank 7N01 substrates in 3.5 wt.% NaCl solution after different time immersion.

Figure 6 shows the polarization curves for the samples with epoxy coatings on the blank 7N01 substrates in 3.5 wt.% NaCl solution after different time immersion. The fitted results are listed in Table 2. For the sample with 1 h immersion in 3.5 wt.% NaCl solution, its corrosion potential (E_corr_) is −0.75 V, and the corrosion current density (I_corr_) is 1.20 × 10^−12^ A/cm^2^. However, for the sample with 168 h immersion, its E_corr_ shifts to −1.15 V, and I_corr_ increases to 2.83 × 10^−9^ A/cm^2^, and a pitting point around −0.80 V can be observed. As for the samples with 192, 240 and 288 h immersion, their E_corr_ are around −1 V, the pitting points are around −0.87 V, and their I_corr_ range from 10^−9^ to 10^−8^ A/cm^2^. These demonstrate that 168 h immersion in 3.5 wt.% NaCl solution causes decline of the corrosion protective property of the epoxy coatings on the blank 7N01 substrates, with the measured E_corr_ shifts from −0.75 V to about −1.1 V and I_corr_ increases by three orders of magnitude from 10^−12^ A/cm^2^ to 10^−9^ A/cm^2^.

**Figure 7:**
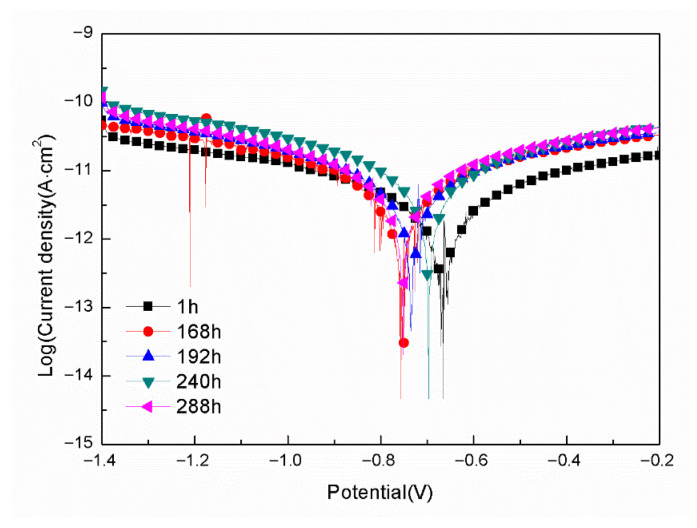
Polarization curves for the samples with epoxy coatings on 7N01 substrates pretreated by 5% BTESPT doped with 5 × 10^−3^ M cerium nitrate and 0.5 g/L zeolite particles in 3.5 wt.% NaCl solution after different time immersion.

Figure 7 gives the polarization curves for the samples with epoxy coatings on 7N01 substrates pretreated by 5% BTESPT doped with 5 × 10^−3^ M cerium nitrate and 0.5 g/L zeolite particles in 3.5 wt.% NaCl solution after different time immersion. The fitted results are listed in Table 3. For the sample with 1 h immersion in 3.5 wt.% NaCl solution, its E_corr_ is −0.67 V, and the I_corr_ is 7.02 × 10^−13^ A/cm^2^. For the samples with 168 to 288 h immersion, their E_corr_ range between −0.76–0.70 V, and I_corr_ range between 2.78 × 10^−12^ to 4.18 × 10^−12^ A/cm^2^. It should be noted that no pitting potential can be observed for all curves. These demonstrate that 168–288 h immersion in 3.5 wt.% NaCl solution causes only a little decline of the corrosion protective property of the epoxy coatings on 7N01 substrates pretreated by 5% BTESPT doped with 5 × 10^−3^ M cerium nitrate and 0.5 g/L zeolite particles.

#### 3.2.2. EIS Tests

**Figure 8:**
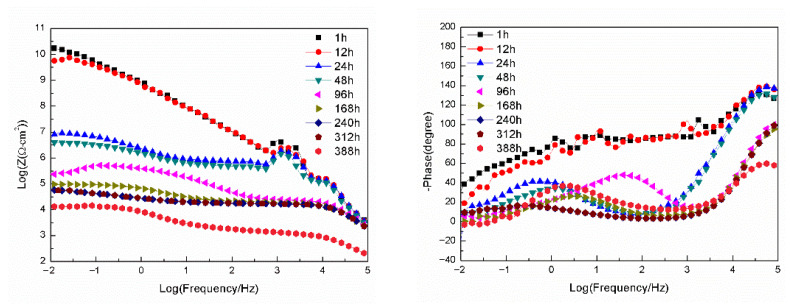
Bode plots of EIS for the sample with epoxy coating on the blank 7N01 substrate in 3.5 wt.% NaCl solution.

Figure 8 presents the Bode plots of EIS for the sample with epoxy coating on the blank 7N01 substrate in 3.5 wt.% NaCl solution. It can be seen that the impedance spectra for 1 h and 12 h shows very high impedance values (about 10^10^ Ω·cm^2^) at 10^−2^ Hz and high phase angles, suggesting that the epoxy coating behaves close to a capacitor. However, the impedance value at 10^−2^ Hz declines rapidly to about 10^7^ Ω·cm^2^ at 24 h. Then, it declines gradually to 1.26 × 10^4^ Ω·cm^2^ as the immersion time prolongs to 388 h, demonstrating that the conductive pathways have been developed in the epoxy coating and the aggressive media can penetrate through the pathways to reach the substrate surface. From the phase-frequency plots, two time constants can be clearly observed after 24 h immersion. The time constant at high frequency is associated with the barrier property of the epoxy coating, and the second one at medium frequency (10^−1^–10^2^ Hz) can be ascribed to the substrate surface oxide film and the interfacial layer between the epoxy and the substrate. It should be noted that for the plots of 96 h and 388 h, an inductive behavior at the lowest frequency can be observed, suggesting occurring of corrosion activity by the aggressive media to surface of the substrate.

**Figure 9:**
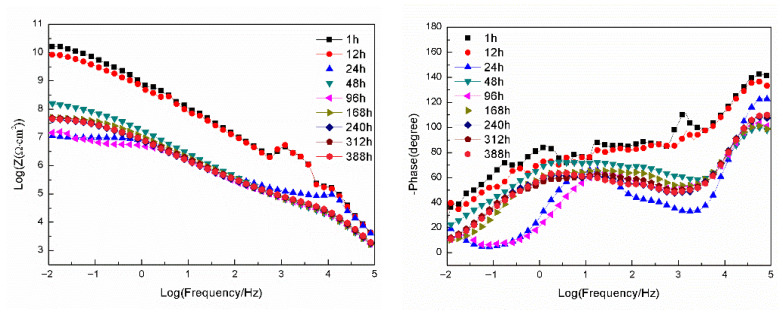
Bode plots of EIS for the sample with epoxy coating on 7N01 substrate pretreated by 5% BTESPT doped with 5 × 10^−3^ M cerium nitrate and 0.5 g/L zeolite particles in 3.5 wt.% NaCl solution.

Figure 9 presents the Bode plots of EIS for the sample with epoxy coating on 7N01 substrate pretreated by 5% BTESPT doped with 5 × 10^−3^ M cerium nitrate and 0.5 g/L zeolite particles in 3.5 wt.% NaCl solution. It can be seen that for 1 h and 12 h, the impedance values at 10^−2^ Hz are about 10^10^ Ω·cm^2^. For the spectrum at 24 h, the impedance value at 10^−2^ Hz declines rapidly to about 10^7^ Ω·cm^2^, which is almost the same as that in Figure 8. This means that the conductive pathways have been developed in the epoxy coating and thus the aggressive media can penetrate through the pathways to reach the composite silane film. For the spectra at 24 h and the following immersion times, two time constants can be observed. The time constant at high frequency is attributed to the barrier property of the epoxy coating, and the second one at medium frequency (10^−1^–10^2^ Hz) can be ascribed to the composite silane film doped with cerium nitrate and zeolite particles. At 48 h the impedance value at 10^−2^ Hz increases, by more than one order of magnitude, to 2 × 10^8^ Ω·cm^2^, which can be ascribed to the barrier property of the composite silane film, especially the action of the doped cerium nitrate and zeolite particles in the film. At 96 h the impedance value at 10^−2^ Hz decreases to 1.5 × 10^7^ Ω·cm^2^, and the phase angle at the lowest frequency increases like that at 24 h, also suggesting reactions of the aggressive media to the composite silane film. From 168 h to 388 h, the impedance value at 10^−2^ Hz remains stable at about 6.2 × 10^7^ Ω·cm^2^, which is more than two to three orders of magnitude higher than that of the corresponding spectra in Figure 8, suggesting that no corrosion occurs to the substrate surface.

### 3.3. Interfacial Microstructure Between Epoxy Coating and Al Substrate After Salt Spray Test

**Figure 10:**
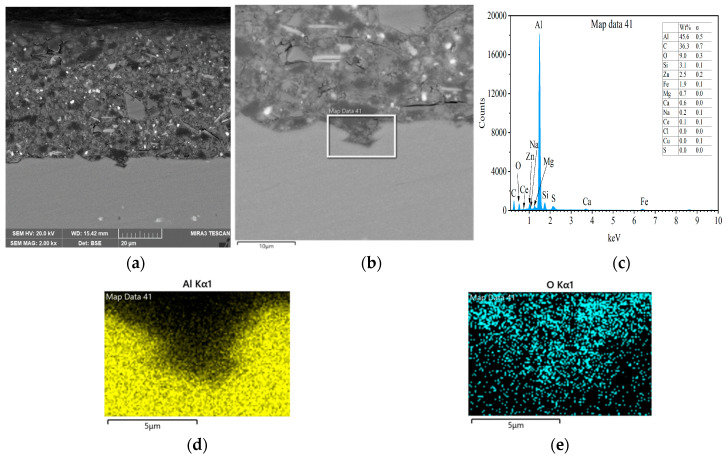
Cross-sectional morphologies and EDS analysis for the sample with epoxy coating on 7N01 substrate after 30 days salt spray test, (**a**) low and (**b**) high magnifications, (**c**) EDS spectrum and mapping of (**d**) Al and (**e**) O in the marked area.

The samples with epoxy coatings after salt spray test for 30 and 60 days were cut and slightly polished to examine the interface between the epoxy coating and the 7N01 substrate. Figure 10 presents the cross-sectional morphologies for the sample with epoxy coating on blank 7N01 substrate and the results of EDS analysis after 30 days salt spray test. Compared to the flat shape without irregularities in Figure 5, 30 days salt spray test changes the interfacial region into apparent irregularity. The results of EDS analysis in the marked region demonstrate that the dark area, the upper part of the marked region, contains relatively low concentration of Al and high amount of O. According to the EIS in Figure 8, at 24 h the aggressive media has penetrated through the developed pathways in epoxy coating to reach at the substrate surface, and after 24 h, the impedance value at 10^−2^ Hz declines gradually to 10^4^ Ω·cm^2^ due to corrosion of the substrate surface by the aggressive media. Therefore, the dark area could be ascribed to the corrosion activity caused by the aggressive media penetrated through epoxy coating to the substrate surface.

**Figure 11:**
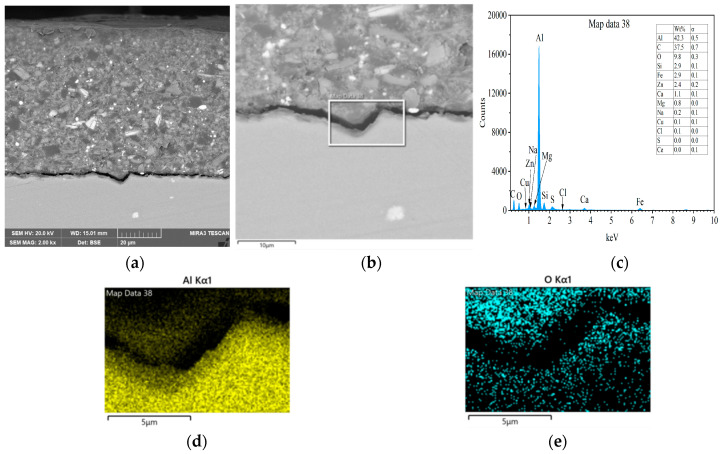
Cross-sectional morphologies and EDS analysis for the sample with epoxy coating on 7N01 substrate after 60 days salt spray test, (**a**) low and (**b**) high magnifications, (**c**) EDS spectrum and mapping of (**d**) Al and (**e**) O in the marked area.

Figure 11 presents the cross-sectional morphologies for the sample with epoxy coating on blank 7N01 substrate and the results of EDS analysis after 60 days salt spray test. It can be seen that after 60 days of salt spray test, the interfacial region is also in irregularity. The results of EDS analysis in the marked region demonstrate that the gray area, the upper part of the marked region, contains relatively low concentration of Al and high amount of O, which is caused by corrosion activity of the aggressive media to the substrate surface. It should be noted that there is a long crack between the epoxy coating and the substrate, which takes the shape of zig-zag. During the salt spray test, the water transports through the epoxy coating to reach the interfacial region, which will cause breaking of Al-O bond and localized dissolution of the oxide layer, and then induce the passage of ions such as hydroxyl, oxygen and chlorine ions inwards and aluminum ions outwards [4,5,6,7,28]. Considering its shape and the EDS mapping of Al and O in the marked area, it could be concluded that the crack is located in the oxide layer formed during the spray salt process underneath the epoxy coating. These features indicate that the substrate endures more severe corrosion after 60 days test than that of 30 days test, as shown in Figure 10.

**Figure 12:**
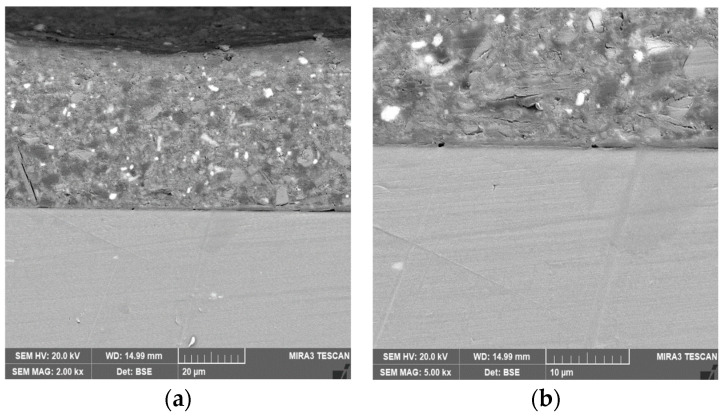
Cross-sectional morphologies for the sample with epoxy coating on 7N01 substrate pretreated by the silane film doped with 5 × 10^−3^ M cerium nitrate and 0.5 g/L zeolite particles after 30 days salt spray test, (**a**) low and (**b**) high magnifications.

Figure 12 presents the cross-sectional morphologies for the sample with epoxy coating on 7N01 substrate pretreated by the composite silane film doped with 5 × 10^−3^ M cerium nitrate and 0.5 g/L zeolite particles after 30 days salt spray test. It can be seen that the interfacial region is still in flat state without apparent irregularities as that of in Figure 5, demonstrating that almost no corrosion occurs to surface of the substrate. According to the EIS in Figure 9, although at 24 h, the aggressive media can penetrate through the developed pathways in epoxy coating to reach the composite silane film, the impedance value at 10^−2^ Hz after 24 h still remains above 10^7^ Ω·cm^2^. Therefore, it could be concluded that it is the composite silane film that avoids penetration of water, oxygen and chloride ions through the film to act on the underneath substrate surface.

**Figure 13:**
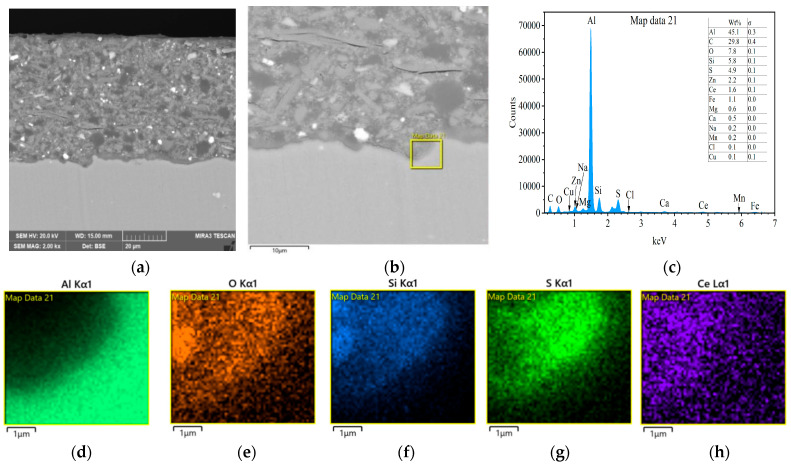
Cross-sectional morphologies and EDS analysis for the sample with epoxy coating on 7N01 substrate pretreated by the silane film doped with 5 × 10^−3^ M cerium nitrate and 0.5 g/L zeolite particles after 60 days salt spray test, (**a**) low and (**b**) high magnifications, (**c**) EDS spectrum and mapping of (**d**) Al, (**e**) O, (**f**) Si, (**g**) S and (**h**) Ce in the marked area.

Figure 13 presents the cross-sectional morphologies for the sample with epoxy coating on 7N01 substrate pretreated by the composite silane film doped with 5 × 10^−3^ M cerium nitrate and 0.5 g/L zeolite particles and the results of EDS analysis after 60 days of salt spray test. Compared to the flat shape in Figure 12, 60 days of salt spray test changes the interfacial region into apparent irregularity. However, no obvious crack like that in Figure 11 can be observed. At the interfacial region, a marked area as shown in Figure 13 b is selected for EDS analysis. The left upper corner of the marked area is dark, which is the newly formed interfacial region by corrosion activity of aggressive media to the Al substrate. The results of EDS analysis demonstrate that it contains a large amount of O, Si, S, and Ce and a low concentration of Al. Elements of S and Ce come from the composite silane film doped with cerium nitrate and zeolite particles, indicating that there is an amount of S and especially Ce distributed in the newly formed interfacial region by corrosion activity. This is different from the case for epoxy coating on blank 7N01 substrate, as shown in Figure 10 and Figure 11, where there are only corrosion products by actions of hydroxyl, oxygen and chlorine ions to the Al substrate. During the salt spray test, the water, oxygen and chlorine ions reached at the interfacial region through the developed pathways in the epoxy coating will diffuse along the interface and lead to growth of the oxide layer and severe corrosion to the substrate surface. While for the case with epoxy coating on 7N01 substrate pretreated by the composite silane film doped with cerium nitrate and zeolite particles, first the water, oxygen and chlorine ions transported through pathways in the epoxy coating are difficult to reach at the interfacial region between the composite silane film and the substrate due to the high barrier effect of the composite silane film; second, the aggressive media, penetrated through the composite silane film, are accompanied by cerium ions released from the silane network and the zeolite particles, which can provide efficiently active protection to the corrosion zones by forming cerium oxide or hydroxide [36,37,38,39,40,41,46]. Therefore, the substrate surface pretreated by the composite silane film endures slight corrosion during the salt spray test.

## 4. Conclusions

In this work, the corrosion performance of epoxy coating on 7N01 substrates pretreated by BTESPT silane solution doped with cerium nitrate and zeolite particles was evaluated. The main conclusions are as follows:

(1) For the samples with epoxy coatings on the blank 7N01 substrates, 288 h immersion in 3.5 wt% NaCl solution causes an increase in I_corr_ from 10^−12^ A/cm^2^ to 10^−8^ A/cm^2^, while for the samples with epoxy coatings on the 7N01 substrates pretreated by 5% BTESPT doped with 5 × 10^−3^ M cerium nitrate and 0.5 g/L zeolite particles 288 h immersion causes only a small increase in I_corr_ from 10^−13^ A/cm^2^ to 10^−12^ A/cm^2^.
(2) For the samples with epoxy coatings on the blank 7N01 substrates, 388 h immersion causes decline of the impedance value at 10^−2^ Hz from 10^10^ Ω·cm^2^ to 1.26 × 10^4^ Ω·cm^2^. While for the 7N01 substrates pretreated by 5% BTESPT doped with 5 × 10^−3^ M cerium nitrate and 0.5 g/L zeolite particles the impedance value at 10^−2^ Hz remains above 10^7^ Ω·cm^2^, more than two to three orders of magnitude higher than the former.
(3) The composite silane film greatly lessens corrosion of the interfacial region between the coating and the substrate. For the samples with epoxy coatings on the blank 7N01 substrates, salt spray test of 30 and 60 days causes the interfacial region from flat into apparent irregularity, and appearance of crack due to severe corrosion activity. While for the substrate pretreated by the composite silane film it endures only slight corrosion.
(4) The thickness of the composite silane film by 5% BTESPT doped with 5 × 10^−3^ M cerium nitrate and 0.5 g/L zeolite particles is about 2.1 μm. Cerium ions are located in the silane film and zeolite particles. The high barrier effect of the composite silane film, and cerium ions released from the silane network and the zeolite particles provide active protection to the substrate surface beneath the epoxy coating.
(5) Future studies could be made to reveal cation exchange of Ce and zeolite particles, evaluate the adhesion strength of the epoxy coating to the substrate and optimize the concentrations of cerium nitrate and zeolite particles in the silane solution.

## Figures and Tables

**Table 1:** The chemical composition (wt.%) of the marked points of the composite silane films corresponding to the EDS spectra in Figure 1.

Point	C	O	Al	Si	S	Ce	Zn	Mg	Cu	Mn	Zr
29	17.56	37.33	5.87	2.69	5.83	28.79	1.04	0	0.15	0	0
36	33.31	26.81	4.06	21.08	8.79	5.12	0.43	0.08	0.01	0.11	0
3	30.53	34.73	0.47	26.55	6.41	0.51	0.31	0.01	0.02	0	0.10
24	19.38	38.76	4.26	27.32	7.44	0.59	0.13	0.12	0.07	0	0.08

**Table 2:** The fitted results of the polarization curves for the samples with epoxy coatings on the blank 7N01 substrates in 3.5 wt.% NaCl solution after different time immersion in Figure 6.

Immersion Time (h)	E_corr_ (V/SCE)	I_corr_ (A/cm^2^)	Pitting Potential (V)
1	−0.75	1.20 × 10^−12^	None
168	−1.15	2.83 × 10^−9^	−0.80
192	−1.09	1.12 × 10^−8^	−0.87
240	−1.04	5.58 × 10^−9^	−0.87
288	−1.10	1.43 × 10^−8^	−0.88

**Table 3:** The fitted results of the polarization curves for the samples with epoxy coatings on 7N01 substrates pretreated by 5% BTESPT doped with 5 × 10^−3^ M cerium nitrate and 0.5 g/L zeolite particles in 3.5 wt.% NaCl solution after different time immersion in Figure 7.

Immersion Time (h)	E_corr_ (V/SCE)	I_corr_ (A/cm^2^)	Pitting Potential (V)
1	−0.67	7.02 × 10^−13^	None
168	−0.76	2.78 × 10^−12^	None
192	−0.73	3.34 × 10^−12^	None
240	−0.70	4.18 × 10^−12^	None
288	−0.75	3.60 × 10^−12^	None

## Data Availability

The original contributions presented in this study are included in the article. Further inquiries can be directed to the corresponding author.
